# Philadelphia County Medical Society

**Published:** 1850-06

**Authors:** Samuel Jackson

**Affiliations:** Late of Northumberland, President of the Society


MEDICAL EXAMINER,

AND

RECORD OF MEDICAL SCIENCE.

NEW SERIES.—NO. LXVI.—JUNE, 1850.

### ORIGINAL COMMUNICATIONS.

PHILADELPHIA COUNTY MEDICAL SOCIETY. *Some Hints on the Comparative Influence of dry and moist hot air in the causation of Disease.* By SAMUEL JACKSON, M. D., (late of Northumberland,) President of the Society.

MR. EDITOR,—I wish to caution the reader of this paper, should it find one, against a great mistake which he may fall into, as did some members of the Society in the discussion thereof. It was argued against me that dry hot summers cause fever. Now this is known to every one, this has become common knowledge, and is true with some exceptions: but mark—though rains are deficient in these summers, the air is not therefore always dry. Streams, millponds, marshes are half and some of them wholly dried up; but there is water enough left somewhere on the earth or under the soil, to moisten the air. With this moisture there is emitted from those half-dried lands, that insensible something called miasma, known only by its effects, as attraction and repulsion, mesmerism, catalysis, &c., are known. But with miasma this paper has nothing to do unless so far as moisture favors the deadly operation thereof; the point to be argued is simply—whether *dry* hot air is not safer than *moist;* and if this be affirmed—whether the washing of pavements and the sprinkling of streets can seriously add to the general moisture.

Much might be added to the paper, but I wish to give it to you precisely as it was read, excepting some matter enclosed in brackets. It was written in haste and in sickness, merely for the purpose of firing the Society's latent heat, and of striking out some scintillations therefrom, by which our lamps might be lighted and our ways made safe. S. J.

GENTLEMEN :—

Our society has appointed a Committee on Hygiene, whose important and laborious province it is, to have regard to the health of this wide-spread, crowded city ; and in order to facilitate their labors I proposed at the last meeting this subject for discussion at the present time—*whether the sprinkling of streets and the washing of pavements is injurious to health?* It may appear yet more appropriate when it is remembered that the city Councils consulted last summer, on this subject, many of our most learned physicians, and that they received very discordant and even contradictory answers. Now it is surely high time that this subject was thoroughly understood, and that physicians were able to answer, not only correctly and philosophically, but with promptness and confidence.

In order to bring the subject fairly before the meeting and to elicit some debate, I have written a few pages which I hope will have this effect. I state it then as a philosophical fact, that *a great excess of heat is tolerated by the human body without injury, provided there be no excess of moisture.* Now I hope the society will not expect me to elucidate this proposition in a very able manner, or even successfully, for I ought not to arrogate much of their time, and, moreover, it is more than probable that much evidence, and even the most weighty, may have entirely escaped me ; but in one point it may be hoped that I shall not be disappointed, my object being to provoke an amicable controversy by which the truth may be fairly and philosophically stated.

With respect to collecting evidence from medical topography, there is much uncertainty, because some unknown elements are supposed by many to conspire with heat and moisture in the causation of disease ; but when health prevails amidst excessive dryness with excessive heat, the evidence is direct and positive as far as human reason can go. One of the strongest facts that can be adduced, is from the travels of Baron De Humboldt. This great man, who had travelled over the whole world either in person or by reading, says, that hot countries are healthy provided they are dry. "The province of Cumana, the coast of Coro, and the plains of Caraccas, sufficiently prove that heat alone is not the cause of great mortality. In countries very warm but at the same time dry, the human race enjoys a longevity perhaps greater than in the temperate zones, where the heat and the climate are excessively variable. Europeans, who at an age a little advanced, migrate into the equinoctial part of the Spanish colonies, generally attain to a serene and happy old age."*

Sir James McGregor in his Medical Sketches, p. 83, says—" for the last eighteen months, in the hot season, the men had much duty. They were daily marched two miles to the fort of Bombay, where they were much exposed to the sun on the garrison duty; and in the heat of the next day, were marched back to their barracks on the island of Colabah. Yet it appears the hot months were the most healthy by far." And speaking of Egypt in the next page he says—" though the degree of heat be very considerably increased, I believe that unless combined with intemperance, it very rarely is the exciting cause of disease."

Dr. Lind says that Senegal, about 16° N. latitude, is healthy during the dry months. The wet season, to use his own words, " is of four months continuance and is the season of sickness; whereas for many months in the dry season, most parts of the country are equally healthy and pleasant with any in the world." Now a portion of this healthy period is when the sun is vertical; for the rains do not set in till he begins to return from the northern tropic. The heat must therefore be excessive, for Lind says that in December, 1763, it was 93° Fahr. " when the sun had made his most distant retreat from that place." What then must it have been under his perpendicular rays towards the latter part of the dry season ?

[South of this region and between the thirteenth and fourteenth degrees of North latitude, the ever lamented Park led forty Europeans more than four hundred miles eastward, from the 26th April to the 10th of June. The heat must have been excessive, for the sun was nearly vertical the whole journey; yet notwith-

* L'Hindoustan et l'Amérique Méridionale. surtout la province de Cumana, la côte de Coro, et les plaines (Llanos) de Caraccas, prouvent assez, que la chaleur seule n'est pas la cause de grande mortalité. Dans les pays très-chauds mais secs à la fois, l'espèce humaine jouit d'une longévité peut-être plus grande que celle que nous observons dans les zones tempérés. C'est le cas partout òu la température et le climat sont excessivement variables. Les Européens qui, à un age un peu avancé, se transportent dans la partie equinoxiale des colonies espagnoles, y parviennent généralement à une belle et heureuse vieillesse. Voy. de Humboldt et Bonpland Trois. part. l. II. p. 61.

standing this and their cruel exposures and hardships and unavoidable irregularities, there was but one death from disease of the climate. One man died of epilepsy and two sickened of dysentery, of whom one recovered though travelling on an ass with a footman on each side to support him on the saddle.

But on the 10th June the rains set in for the wet season and a tornado says Mr. Park—*Journal of Second Mission*—" had an instant effect on the health of the soldiers and proved to us the *beginning of sorrow.* I had proudly flattered myself that we should reach the Niger with a moderate loss; two men had been sick of the dysentery of whom one had recovered completely on the march, and the other would doubtless have recovered had he not been wet by the rain. But now the rains had set in and I trembled to think that we were only half way through our journey."

The next day, twelve men were sick; all the rest sickened in a short time; and before the raining season was over, they were all dead except four, though Park was an educated experienced physician and provided with bark and other medicines.

Such is the deadly change effected by adding moisture to heat. Now let us see how quickly this fatal moisture is dried up and health restored. In Park's Journal of his first travels, we find chapter twenty begins with the following notable paragraphs:

" The whole of my route both in going and returning, having been confined to a tract of country bounded nearly by the twelfth and fifteenth parallels of latitude, the reader must imagine, that I found the climate in most places extremely hot; but nowhere did I feel the heat so intense and oppressive as in the camp at Benowm, of which mention has been made in a former place. In some parts, where the country ascends into hills, the air is at all times comparatively cool; yet none of the districts which I traversed could properly be called mountainous. About the middle of June, the hot and sultry atmosphere is agitated by violent gusts of wind, called *tornadoes*, accompanied with thunder and rain. These usher in what is denominated *the rainy season*, which continues until the month of November. During this time the diurnal rains are very heavy, and the prevailing winds are from the south west. The termination of the rainy season is likewise attended with violent tornadoes; after which the wind shifts to the north-east, and continues to blow from that quarter during the rest of the year.

" When the wind sets in from the north east, it produces a wonderful change in the face of the country: the grass soon becomes dry and withered, the rivers subside very rapidly, and many of the trees shed their leaves. About this period is commonly felt the *harmattan*, a dry and parching wind, blowing from the north-east, and accompanied by a thick smoky haze, through which the sun appears of a dull red color. This wind, in passing over the great desert of Sahara, acquires a very strong attraction for humidity, and parches up everthing exposed to its current. It is, however, reckoned very salutary, particularly to Europeans, who generally recover their health during its continuance. I experienced immediate relief from sickness, both at Dr. Laidley's and at Kamalia, during the harmattan. Indeed, the air during the rainy season is so loaded with moisture, that clothes, shoes, trunks, and every thing that is not close to the fire, become damp and mouldy, and the inhabitants may be said to live in a sort of vapor bath, but this dry wind braces up the solids, which were before relaxed, gives a cheerful flow of spirits, and is even pleasant to respiration."]

Bordering on this region is the great Sahara, where heat and drought prevail to an excessive degree, and yet the wandering Arabs are so healthy, that they find it more difficult to die than other nations find it to live. They endure to a greater age than any other people, and when at last they die, it is not of disease, but rather of a natural mummification. So dry and hot is the air, that their dead bodies, as well as their camels flesh, dry up and resist putrefaction.

Adjoining Sahara lies the ever celebrated land of Egypt: consider how healthy this country must have been in ancient times, when its little delta with its mere strip of land up the Nile, supported an immense population and furnished such a surplus of corn that it was called the granary of the Roman empire. Here it seldom rains and the lands are dried by the scorching winds from the Libyan desert, yet it was and continues to be a healthy country, the plague excepted. Even this is arrested by drought and heat. Volney says that in Egypt, the winter, being warm and humid, foments the plague ; summer, being hot and dry, destroys it. Summer acts on the plague as it does on flesh which it prevents from putrefying." Heat, he continues, "is injurious only when combined with moisture."* In accordance with this, Sir J. McGregor says p. 99,

* En Egypte, l'hiver fomente la peste parce qu'il est humide et doux ; l'été la détruit parce qu'il est chaud et sec. Il agit sur elle comme sur les viandes qu'il ne laisse pas pourrir. La chaleur n'est malfaisante qu' autant qu' elle se joint à l'humidité." *Voy. en Syrie et en Egypte, vol. I.* 231.

that "the diseases which occurred in the army of Egypt, were not numerous."

Sonnini declares that the modern Egyptians have neglected every thing that could contribute to the health of their country, and have done every thing to render it unhealthy. He says "under the hands of these barbarians, the ancient works indispensable to the fertility of the soil and salubrity of the air, were daily disappearing. Marshes occupied the place of useful lakes: some canals were filled up, others were nothing but a body of stagnant water during one part of the year, the fetid effluvia of which were disseminated far around. The stench arising from the carcases of dead animals infested the country and sometimes even the precincts of the towns. In short it seemed as if the inhabitants studied to render their country unhealthy. What opinion then, says he, must we entertain of the goodness of a climate which, in spite of the mischiefs of careless ignorance, had acquired no dangerous influence." He says much more in praise of the climate and adds, that "you may see Turks from Constantinople, worn out by debauchery and disease, who after a little stay in this country resume the appearance of health."—*Hunter's Translat.*

But as already observed, the examples from medical topography in favor of dryness, are liable to much contradiction or caviling, therefore we should be glad to find some that might prove unobjectionable. A strong fact in favour of a dry atmosphere, is found in *Combe on the Constitution of Man*, one of the best and most useful books in relation to health and morals that ever saw the light. In the first edition, and we presume in all subsequent ones, there is a letter from Captain Murray of the Royal Navy, on the subject of preserving the health of his men. His ship sailed from Plymouth to the West Indies in December 1823, having just returned from the coast of Labrador and Newfoundland, where she had been stationed two years, the crew amounting to 150. Here was a sudden and dangerous transition from excessive cold to excessive heat. Now after detailing his care with respect to the procuring of flannel shirts for every man and the compelling of his men to wear them, he says—"the quarter and main decks were scrubbed with sand and water and wet holystones every *morning at daylight.* The lower decks, cockpit and storerooms were scrubbed every day after breakfast with *dry* holystones and *hot* sand until quite white, the sand being carefully swept up and thrown overboard. The pump-well was also swabbed out dry and then scrubbed with holy stones and *hot* sand; and here, as well as in every part of the ship which was liable to damp, brodiestoves were constantly used until every appearance of humidity had vanished. The lower deck and cockpit were washed once every week in dry weather, but brodiestoves were kept constantly burning in them until they were dry."

"I was employed on the coast of Caraccas, the West India islands, and gulf of Mexico; and in course of service, I visited Trinidad, Margarita, Cocha, Cumana, Nueva Barcelona, Laguira, Porto Cabello, and Maracaibo; all the West India islands from Tobago to Cuba both inclusive; as also Curaçoa, and Druba, and several of those places repeatedly; also Vera Cruz and Tampico; which you must admit must have given a trial to the constitutions of my men after two years among the icebergs of Labrador, without a summer intervening between that icy coast and that of Caraccas; yet I arrived in England June 24th without having buried a single man or officer belonging to the ship, or indeed having a single man on the sick list; from which I am satisfied that *a dry ship will always be a healthy one in any climate.* When in command of the Recruit of 18 guns, in the year 1809, I was sent to Vera Cruz where I found the —— 46, the —— 42, the —— 18, and —— gun-brig; we were joined by the —— 36, and the —— 18. During the period we remained at anchor, from eight to ten weeks, the three frigates lost from thirty to fifty men each, the brigs sixteen to eighteen, the —— lost most of her crew with two different commanders; yet the Recruit, although moored in the midst of the squadron and constant intercourse was held with the other ships, did not lose a man and had none sick. Now as some of these ships had been as long in the West Indies as the Recruit, we cannot attribute her singularly healthy state to seasoning, nor to superior cleanliness . . . . . . perhaps it may be attributed to cheerfulness in the men; to my never allowing them to go on shore in the morning on an empty stomach; to the use of dry sand and holystone for the ship; to never working them in the sun; perhaps to accident. Were I asked my opinion I would say, that cheerfulness contributes more to keeping a ship's company healthy than any precaution that can be adopted."

But whence this salutary cheerfulness? Was it not principally the result of their continual health? Would they not have been dispirited, had they seen their mates whelmed in the ocean as they saw the men from the other ships? What then was the source of this unexampled health? Captain Murray says—"*I am satisfied that a dry ship will always be a healthy one in any climate.*"

Though the experiments of Dr. Fordyce and Sir Charles Blagden are not entirely satisfactory in all their details, yet there is a fact of importance that was fairly established by them:—they could remain much longer in a *dry* heated chamber than in a *moist* one; a fact which no one can doubt who has known the difference between a northerly wind in summer and the *madidus auster.*

Does any one object that the winds on and from the deserts of Asia and Africa, are so dry as not only to prove injurious but sometimes suddenly fatal, he may be answered, that I am not proposing to defend a *quid nimis.* While we propose the heat of our own summers as not hurtful *in itself*, I would yet deprecate the Harmattan or Samiel wind; so while others would defend a greater degree of moisture than I could approve, they would yet avoid the blasts of the moist Sirocco.* Whoever has the practice of crying *ne quid nimis*, should beware that he has definite notions of what he is deprecating. Rooms warmed by stoves and furnaces do certainly require an evaporation of water, particularly in dry and freezing weather, for the air is then almost destitute of vapor and when heated by mineral coal, it acts on the lungs by carrying off too much fluid: so also the air is too dry at great elevations for it dessicates the lungs; but there is a degree of dryness which is propitious both to health, comfort, and exertion. On the high table land of Mexico the Americans fought like lions, under a sun at that time nearly vertical, and it is almost incredible what exertions they made. Some of them, in relating to me their inordinate labors, remarked, that they did not sweat; this was a great mistake, for their life depended on this salutary function. The sweat was carried off by the dry but sufficiently dense air of that moderate elevation, so fast as not to be perceived by them. So

* Est modus in rebus, sunt certi denique fines
Quos ultra citraque nequit consistere rectum.
*Horat. Sat.* 1.

Schmidtmeyer says, " that in Chili, notwithstanding the very high temperature in summer, the perspiration passes off so entirely in the insensible form, that during the most violent exercise, it might be doubted whether any exist." *Dunglison's Hyg. Ch. I.* At Mexico we heard of no one dying of mere heat, but at the battle of Monmouth, which was fought in a low country not far from the sea, June 28th, 1778, both English and Americans, though they fought in the old fashioned and more gentle way, fell by dozens, perishing of mere heat, without the stroke of an enemy.

It is far more difficult, however, to render probable the salubrity of hot dry air, than to prove the perniciousness of hot air that is loaded with vapour; for look into authors and you will find that this is mentioned by every one as influential in causing or promoting the causes of disease. Pringle would appear from a short passage to contradict this when he says, *Part II. Ch.* 2, " pestilential diseases have frequently occurred in dry and hot summers, and I have observed that the most sickly seasons in the field have been attended with the greatest heat and the least rain." But here he forgets to tell his whole story at once, for the troops did not sicken *till they were encamped in the marshes*, as may be seen in the same chapter where he makes this assertion which he nowhere contradicts :—" in every campaign, he says, no epidemic ever ensued on the greatest heats till the perspiration was stopped by wet clothes, wet beds, dews, or fogs : and in the last campaign, though the heats were great, yet they were the cause of little sickness till the troops were *cantoned in marshes*." By pestilential diseases, moreover, he means the plague, for he refers to Diermb. and Bacon on this disease; and that dry heat does not favor this we have various testimony. Lind says it ceases in Egypt the beginning of June, " when the periodical hot winds from the deserts of Nubia and Ethiopia have rendered the air pure and wholesome :"—not the overflowing of the Nile, as some have ignorantly asserted, for this has not yet taken place. The same ceasing of the plague in hot dry weather, we mentioned above from Volney ; and Sonnini says, *Hunter's translation*, *p.* 18, that it always ceases at the summer solstice.

But it often happens that the cautious opinion of an authoritative man is of more weight than a multitude of facts, from which every one who pleases, may often find ways of escape. With respect then to the operation of an excessive moisture, I presume there is no better authority than the author of a paper in the Philadelphia Medical and Physical Journal, vol. xi. 297, a learned and eminent writer, who has devoted much time to this and similar studies.

"Simple atmospheric moisture, without any great extreme of temperature, is not found to be productive of bad effects on a healthy subject conforming to the rules of hygiene. It gives, however, greater activity to the operation of both heat and cold; and is, when conjoined with either, a frequent cause of disease. Humidity acts on the skin by diminishing exhalation and absorption, and producing an atony of the extreme vessels. The circulation participates in this languor. The thirst is less, and the secretion of urine more abundant. Respiration is laborious; a kind of oppression is felt, owing probably to the watery vapor not being completely expelled from the lungs; the air which served for inhalation having been already charged with humidity. The secretion from the mucous membranes is usually augmented, hence a species of coryza and even diarrhœa. The senses are usually more obtuse in a humid air, owing to the undue moistening of their surfaces. Sensibility in general is blunted, muscular power diminished, and all the movements of the body slower and less agile.

"Tourtelle in his Hygiene, assures us that a humid state of the atmosphere is unfavourable to vegetable as well as animal life, and alters strangely the fluids and secretions. In a wet spring the flowers of the yellow laurel rose, (ægolethron,) are poisonous, and the honey which the bees extract from them has similar noxious properties.

"Moisture joined to cold constitutes one of the most efficient agents in the production of scurvy, scrofula, cynanche tonsillaris and trachealis, catarrhal fevers, and rheumatism. It is often the exciting cause of diarrhœa, dysentery, and tetanus, the more especially if there has been preceding great heat. In northern latitudes there is no condition of the air so invariably pernicious, so chilling and oppressive to the organs of respiration as the combination of frost with fog. It is this state, which has been found to accompany, if not produce, extensive influenzas and wide spreading pneumonic diseases.

"In southern countries on the other hand, the union of heat and moisture may be viewed as exerting an influence paramount to that of all other causes, whether it be between decks of a ship, in close barracks, or in the lower parts of a city, and the swamps and low grounds in the country. This condition of the atmosphere acquires greater intensity of effect from the calms, or little circulation of air so often accompanying it. There is in fact a stagnant atmosphere which needs no miasmatic aid to give it destructive power. Moisture and cold, will, if ventilation be not attended to, produce scurvies on board a ship. Moisture and heat, with equal negligence in ventilation, will, in the same situation, be followed by remittent or gastric fevers."

So strongly was the author, Dr. John Bell, impressed with the dangers from an excess of moisture, that he considers it when conjoined with heat, as one cause of what have been called koino-miasmatic fevers. This, moreover, has been the opinion of other philosophers. You are aware that Dr. Willis and Mr. Hopkins lately excogitated a theory of the causation of fever in which they considered hot air saturated with vapor as the sole remote cause, thus annihilating all the old imaginations of miasma. Though far from adopting their doctrine, yet I am fully persuaded that these gentlemen have reasoned fairly concerning the operation of vapor on the human body.

They say it is comparatively easy to remain, for a certain time, in a stove room of a temperature considerably higher than that of the body, *if the air be dry*. Here then is a free perspiration and a rapid solution of it by the parching air. Thus M. de la Roche, Dr. Willis says, found that animals lived longer in dry air "*of excessively high temperature*" than in a moist air of their own temperature. Now, he continues, it is highly interesting to observe, that the air of unhealthy intertropical climates differs little from that of a vapor bath, at 80° or 90° of Fahr. The dew point is very high and the air already nearly saturated with water, cannot dissolve and carry off the perspiration; the patient therefore becomes shut up in a little atmosphere of his own which excludes a due proportion of oxygen and prevents perspiration—thus the functions are perverted and fever, as they say, may be generated.

Now independently of the direct operation of heat and moisture, whatever that may be, the common experience of all men agrees, that the combination is highly favorable to the generation and spreading of disease. This consideration ought to weigh heavy with the guardians of public health. Whether the streets of Philadelphia are in winter or summer too damp, hence depressing the vital forces and generating or spreading diseases, is a question we ought to be prepared to answer with confidence and truth. That this is the case from October to May inclusive, at least whenever the atmosphere is not rendered anhydrous by continued severe frost, hardly any one can answer in the negative; but with respect to the hotter portions of the summer months, a doubt has been expressed by many.

It is observed by Cowper that God made the country and man the cities ; by which he means that cities are like their inhabitants, mere productions of degenerate beings and partakers of all their evils. What physical causes then are found in a great city which are not common to it and to country situations, *both equally favored by nature?* It may be fairly answered that dampness is one of the most prominent. You cannot spare me time to enter into proofs of this, nor do I suppose that proofs are necessary. It is felt in all the temperate months and in all the cold months when vapour is not locked up by frost : it is perceived in the hot months, for no sooner do we go beyond the limits of the city in August or September than the invigorating influence of the country air is sensibly felt; no sooner do we return to the city than we perceive the depressing spirit of dampness. What is it that produces more disease, and particularly cholera infantum, in damp sunless alleys and courts than in the open streets ? What produces more in the open streets than in the country ? Why do you hurry the little half-dead patients into the country air ? Do you say, it is *impure* and not *damp* air that you fly from ? Be it so : but as dampness is a mortal propagator of foulness and all the remote causes of disease, and is often an exciting cause also, do not neglect, let us say to the Councils, to obviate it by all the means in your power.

Whether the sprinkling of streets and the watering of pavements, are sufficient to increase the manifest evil, ought to be well considered ; for if all the principal streets were traversed daily by the great sprinkling machine, there would surely be a few degrees added to the hygrometric state of the atmosphere. But take this in conjunction with the general washing of the pavements, side alleys, and yards, and who can doubt, but that a sensible addition will be made to the existing vapor, already too great both in winter and summer ? Let us remember too, that over these pavements deluged with water and rendered damp very often till they are deluged again either from the clouds or the hydrants, our most delicate and sickly ladies are accustomed to walk for their health.

The sprinkling of the streets has been compared to a shower of rain in its pleasant and salutary effects, but the comparison is utterly unjust. Rain is pure water from the higher regions and in its descent, it washes and purifies the air; but the sprinkling is done with the impure and pestiferous water of the Schuylkill. The water from the machine, were it pure, could not wash the air, as it has not twenty-four inches to fall. Rain, moreover, not only washes the impure air through which it descends, but, as it consists of vesicles of pure water containing the pure air from the higher regions, it supplies this "*pabulum vitæ*" to no inconsiderable degree.

Does any one say that chemistry has often been applied and has found in the Schuylkill water nothing pernicious, let him be answered that chemistry is not a science but a cunning art. 'Tis true, it has done wonders for medicine, for the arts, and for philosophy, but still it must be considered as imperfect and fallacious. There are many impurities and poisons in the air which it cannot ascertain, and why should there not be in water, particularly when our olfactory organs are offended thereby? I have walked in the rear of the machine and never without perceiving a very offensive smell, which with the vapour and the consequent exclusion of oxygen, rendered respiration oppressive and walking laborious. I have heard others make the same complaint whose lungs like my own, are perfectly sound. Those fortunate persons who can ride in their carriage, need less breath and of course they are less annoyed.

Will any one say that he has stood at the Franklin-square fountain without perceiving a heavy and oppressive fetor? I need not name the various sources of impurity in the Schuylkill water, but there is one I may mention which perhaps is not generally known. A gentleman of great learning and authority told me, that he stood by one of the Fairmount fountains when the water had been nearly drawn off, and that he saw thousands of fish floundering in the bottom. He added that the various excrements of these were breathed in our streets and drunk at our tables; for when he went to the leeward of the fountain, their smell was intolerable. A medical professor of great learning thinks that he perceives a foul exhalation from the water pans of the furnaces by which houses are warmed: that from day to day fresh water is added without washing the pan and the foulness of the sediment rises in distillation.

Some one may repeat what I have often heard, that this water must make nearer approaches to purity than that of the Thames which in part supplies London ; but this comparison does not purify the Schuylkill. True, it is better to have water from this river than from the Thames or from our own pumps, so it is better to have yellow fever than cholera: but when we cannot avoid an evil, let us be honest and not persuade ourselves that it is a good ; let us not deceive ourselves into the sluggard's optimism which is an enemy to all progress. The smell of the Schuylkill water when poured forth in large quantities so as to be diffused and dissolved in the air, is truly very offensive; nor can you say that the bottom of your pitcher in the morning is altogether inodorous. When I stood by the fountains in New York and Boston, though they poured forth water much faster than ours, I perceived no smell. The time will no doubt come when, from causes unnecessary to name, Philadelphia will be obliged to bring her water from the Lehigh or the Delaware far above the Falls.

This impure and odorous water then is showered over the streets, where, mingling with the impure miasma-genetic materials there spread abroad, a poisonous exhalation is diffused through the air, is precipitated by the dew of the coming night, and breathed in its descent by all who would enjoy the external air whether in the houses or streets.

Should it be inquired—would you not wash out the gutters and sprinkle the streets while the scavengers are engaged in scraping and sweeping—do both with all my heart, for this is a very different affair. Let the waters pour down the gutters in the greatest abundance, for it passes on rapidly in a narrow stream and has not time to evaporate ; and the scavengers sprinkling is raked into heaps and hauled away, before there is time to do mischief.

What then is to be done with the filthy pavements ? Sweep them once a day and they will not be filthy—they will be clean enough for any queen in Europe, even for her who walked over the mud on Raleigh's ermine. If the *aristoi* wish to distinguish themselves, let them use dry sand, as in Ancient Rome they used sawdust for such purposes. Juvenal says to his friend—" wretched man, are you in a state of hurry and alarm, lest your hall and your porch covered with mud, may displease the eyes of your coming friend? A little servant with a half bushel of saw-dust will soon cleanse them. But are you not concerned, that your son may see his home sanctified, unspotted, and free from vice?"*

The washing of the pavements is a very great evil whether in winter or in summer, independently of the dampness it adds to the atmosphere. If done in the morning, it annoys passengers by wetting their feet, and not unfrequently the pavements become thereby covered with ice and dangerous to all pedestrians: if done in the evening as it often is, it is sure to have this effect in freezing weather; and in the milder, they remain wet till the next day noon. Now it is a fact, that there are many sick people who cannot take fresh air except by walking, and to them, and indeed to all delicate people, the damp pavements present a very formidable obstacle. This is not an hypothesis of mine, but a stubborn fact from the lips and hearts of many sickly people.

*Ergo, miser, trepidas, ne stercore fœda canino
Atria displiceant oculis venientis amici,
Ne perfusa luto sit porticus; et tamen uno
Semodio scobis hæc emundet servulus unus.
Illud non agitas, ut sanctum filius omni
Aspiciat sine labe domum vitioque carentem?—JUV. xiv. 64.

